# Supplementary material for: Hematuria Is Associated with More Severe Acute Tubulointerstitial Nephritis
Source: J Clin Med. 2020 Jul 7;9(7):2135. doi: 10.3390/jcm9072135 (PMC7408949; doi:10.3390/jcm9072135)
Supplement: Supplementary file 1 [file jcm-09-02135-s001.pdf]

Table S1. Acute kidney injury secondary to ATIN according to treatment with steroids.

Variables	No steroids (*n* = 20)	Steroids (*n* = 90)	*p*-Value
Onset serum Cr (mg/dL)	3.29 ± 2.10	3.74 ± 3.64	0.59
Peak serum Cr (mg/dL)	5.02 ± 2.54	5.05 ± 4.09	0.97
Biopsy serum Cr (mg/dL)	2.93 ± 1.56	3.70 ± 2.76	0.23
Biopsy UPCR (mg/g)	816.80 (398-1354)	708.00 (321-1121)	0.61
End of follow-up serum Cr (mg/dL)	2.99 ± 2.46	2.61 ± 2.45	0.52
Other characteristics			
Eosinophilia, *n* (%)	4 (20)	28 (31.1)	0.27
Eosinophil number (x10^3/µL)	0.29 ± 0.14	0.35 ± 0.25	0.13
CRP (µg/L)	38.76 ± 76.06	48.98 ± 70.84	0.57
Asymptomatic, *n* (%)	0	4 (4.4)	0.33
Proteinuria, *n* (%)	14 (70)	71 (78.9)	0.47
Nephrotic syndrome, *n* (%)	1 (5)	1 (1.1)	0.34
Hypertension, *n* (%)	12 (60)	46 (51.1)	0.63
AKI, *n* (%)	20 (100)	85 (94.4)	0.55
Acute dialysis, *n* (%)	2 (10)	6 (6.7)	0.62

UACR: urinary albumin:creatinine ratio; CRP: C reactive protein; AKI, acute kidney injury.

Table S2. Outcomes according to the use of steroids.

Variables	No steroids (*n* = 20)	Steroids (*n* = 90)	*p*-Value
Renal replacement therapy, *n* (%)	2 (10)	8 (8.9)	0.85
Recovered kidney function, *n* (%)	3 (15)	37 (41.1)	0.12
Time to recover renal function, days	352.50 (82-915)	60 (30-142)	0.25
Relapse, *n* (%)	0 (0)	7 (7.8)	0.21

Table S3. Renal outcomes according to proteinuria.

A. Outcomes according to proteinuria (highest vs. lowest tertile of proteinuria).

Variables	Highest Tertile (*n*=22)	Lowest Tertile (*n*=22)	*p*-Value
End of follow-up serum Cr (mg/dL)	3.55 ± 3.22	2.84 ± 2.42	0.42
Renal replacement therapy, *n* (%)	5 (22.7)	0	**0.03**
Recovered kidney function, *n* (%)	7 (31.8)	7 (31.8)	1.00
Time to recover renal function, days	83 (45-90)	30 (17-240)	0.48
Relapse, *n* (%)	0 (0.0)	3 (18.2)	0.07

B. Outcomes according to proteinuria (proteinuria yes/no (>150 mg/g of urinary creatinine or >250 mg in 24 h).

Variables	No proteinuria (*n*=7)	Proteinuria (*n*=59)	*p*-Value
End of follow-up serum Cr (mg/dL)	4.13 ± 3.73	3.07 ± 2.81	0.27
Renal replacement therapy, *n* (%)	1 (14.3)	7 (11.9)	0.93
Recovered kidney function, *n* (%)	2 (28.6)	21 (38.2)	0.62
Time to recover renal function, days	161	45 (23-105)	0.19
Relapse, *n* (%)	1 (14.3)	4 (5.1)	0.50

C. Outcomes according to proteinuria (proteinuria above and below the median value).

Variables	Above Median (*n*=33)	Below Median (*n*=32)	*p*-Value
End of follow-up serum Cr (mg/dL)	3.21 ± 2.95	3.20 ± 2.95	0.67
Renal replacement therapy, *n* (%)	6 (18.2)	2 (6.3)	0.19
Recovered kidney function, *n* (%)	13 (41.9)	10 (33.3)	0.49
Time to recover renal function, days	67.50 (34-90)	40 (17-210)	0.58
Relapse, *n* (%)	4 (12.1)	0	0.03
